# Molecular Dynamics Studies of the Inhibitor C34 Binding to the Wild-Type and Mutant HIV-1 gp41: Inhibitory and Drug Resistant Mechanism

**DOI:** 10.1371/journal.pone.0111923

**Published:** 2014-11-13

**Authors:** Xueting Ma, Jianjun Tan, Min Su, Chunhua Li, Xiaoyi Zhang, Cunxin Wang

**Affiliations:** College of Life Science and Bioengineering, Beijing University of Technology, Beijing, China; Chinese Academy of Medical Sciences, China

## Abstract

Mutations on NHR (N-terminal heptad repeat) associated with resistance to fusion inhibitor were observed. In addition, mutations on CHR (C-terminal heptad repeat) accompanied NHR mutations of gp41 are noted in many cases, like N43D/S138A double mutation. In this work, we explored the drug resistant mechanism of N43D mutation and the role of S138A second mutation in drug resistance. The binding modes of the wild type gp41 and the two mutants, N43D and N43D/S138A, with the HIV-1 fusion inhibitor C34, a 34-residue peptide mimicking CHR of gp41, were carried out by using molecular dynamics simulations. Based on the MD simulations, N43D mutation affects not only the stability of C34 binding, but also the binding energy of the inhibitor C34. Because N43D mutation may also affect the stable conformation of 6-HB, we introduced S138A second mutation into CHR of gp41 and determined the impact of this mutation. Through the comparative analysis of MD results of the N43D mutant and the N43D/S138A mutant, we found that CHR with S138A mutation shown more favorable affinity to NHR. Compelling differences in structures have been observed for these two mutants, particularly in the binding modes and in the hydrophobic interactions of the CHR (C34) located near the hydrophobic groove of the NHR. Because the conformational stability of 6-HB is important to HIV-1 infection, we suggested a hypothetical mechanism for the drug resistance: N43D single mutation not only impact the binding of inhibitor, but also affect the affinity between NHR and CHR of gp41, thus may reduce the rate of membrane fusion; compensatory mutation S138A would induce greater hydrophobic interactions between NHR and CHR, and render the CHR more compatible to NHR than inhibitors.

## Introduction

Human immunodeficiency virus type 1 (HIV-1) is the causative agent of the acquired immunodeficiency syndrome (AIDS). The envelope glycoprotein is involved in HIV-1 infection. It is composed of two noncovalently binded subunits, gp120 and gp41. The transmembrane subunit gp41, a 345 residues glycoprotein encoded by the *env* gene, is composed of three distinct functional domains: a cytoplasm domain (residues 705–856), a transmembrane domain (TM) (residues 684–705) and an extracellular domain. The extracellular domain contains a NHR (N-terminal heptad repeat) motif and a CHR (C-terminal heptad repeat) motif and every three extracelluar domains could associate to form a trimer-of-hairpins. In this trimer-of-hairpins structure, the NHR domain forms a central trimeric coiled coil which is surrounded by (three C-peptide regions) CHR domain at hydrophobic grooves of the N-trimer. A conformational transition of trimer-of-hairpins is involved in the membrane fusion mediated by gp41. Before the fusion, the NHR domain and CHR domain composed a pre-hairpins, which is supposed to be an extended intermediate structure. After the conformational transition, the CHR domain is compressed into α-helix along with the inner trimeric core of the NHR. Ultimately, the trimer-of-hairpins is formed, and it is also called as six-helix bundle (6-HB) [1], [2]. This 6-HB is essential for the process of HIV-1-mediated membrane fusion,since the NHR domain is proximal to the fusion peptide (FP) which is inserted into the target cell membrane, and the CHR domain is adjacent to the trans-membrane region of HIV-1 [3].

Fusion inhibitors is reputed to block the formation of 6-HB effectively. Among which, SJ-2176 is the first potent anti-HIV-1 peptide which derived from the CHR domain of gp41. SJ-2176 could inhibit the membrane fusion at nanomolar level [4]. After SJ-2176, many peptides derived from NHR domain or CHR domain come up, such as N51 (HIV-1 HXB2 540–590), N36 (HIV-1 HXB2 546–581), N34 (HIV-1 HXB2 546–579), C43 (HIV-1 HXB2 624–666), C34 (HIV-1 HXB2 628–661) and C28 (HIV-1 HXB2 628–655) [5]–[7]. ENF is the first fusion inhibitor that licensed for marketing as antiretroviral drug. It is homologous to gp41 as it mimics 36 amino acids of CHR domain. Recently, it has presented a class of peptide fusion inihibitors with enhanced inhibitory activity. Among them, sifuvirtide (SFT) is a potent inhibitor that has entered the clinical trial stage. It contains 36 amino acids covering part of sequences and structures of native gp41 CHR [8], [9]. D-peptides, which has shown several potential advantages has the capability to become a new class of fusion inhibitors. Its advantages include: resistance to proteolytic degradation, prolonged half-life in serum and reduced immunogenicity in circulation, and possibility for oral administration [10].

Now,the major problem associated with HIV-1 fusion inhibitors is molecular resistance. Fusion inhibitors always lose their effect due to rapid point mutations of the genome of HIV-1. Common mutations have been identified on 6-HB of gp41 and these mutations could attenuate binding affinity and sensitivity of fusion inhibitors in vitro [11]–[13]. However, in return (or “on the other hand”), these mutations would have unfavorable effects on viral infectivity through interfering with interaction between viral HR1 and HR2 domains [14]. The GIV motif of NHR (L33 to L45) is initially identified ENF (the first fusion inhibitor to prevent the entry of HIV-1) dependent on HIV-1 variant mutation site [15], [16]. Now, many mutations are identified, single mutations like G36D/S, V38A/M, Q40H, N42S/T/D/E, N43D/K/S, L44M and L45M have been generally showed to reduce the sensitivity of ENF [12]. Mutations on gp41 of HIV-1 is thought to reduced the affinity between inhibitors and gp41 by disrupting packing, affecting the electrostatic, hydrophobic interactions [17]. However, when single mutations are introduced on NHR or CHR, these mutations are likely to impact the viral entry: reduce the rate of membrane fusion [18]; affect the pathogenicity, replicative fitness, immunological recognition,infectivity of viral [14], [19]; render the HIV-1 more sensitive to neutralizing antibodies [14], [18]. It has been thought that mutations in NHR are harmful to envelope processing and HIV-1 fusion [20]–[22]. Some studies indicate that single mutation on NHR of gp41 would induce the double compensatory mutation on CHR which may compensate those defects and restore the function of gp41 [23], [24].

The aims of this work is to: 1, explore the changes on energy and structure between wild type and mutation type(N43D) of gp41; 2, explore the role of CHR compensatory mutation S138A by energy and structural analysis; 3, hypothesize mechanisms of drug resistance induced by N43D single mutation and N43D/S138 double mutation.

## Methods

### System Preparation

The ligand structure C34, receptor structures were obtained from the Protein Data Bank (PDB code 2XRA) [25]. This PDB present the crystal structure of the HK20 Fab in complex with a gp41 mimetic 5-Helix, we selected 5-Helix which contains three NHR domain and two CHR domain of gp41 as receptor. The mutations of N43D in receptor and S138A in ligand C34 were constructed by the Biopolymer module in Sybyl, version 7.3 (Sybyl Molecular Modeling Software, Tripos Associates, Inc., St. Louis, MO, USA). The wild type gp41 5-Helix receptor was denoted as WT, the 5-Helix with N43D mutation was named as N43D. All hydrogen atoms were added with xLeap program according to the amber ff03 force field. Every structure was immersed in a water box filled with the TIP3P water molecules, keeping a minimum distance of 10 Å between the solute and each face of the box. The proper type and number of counterions (Na^+^ or Cl^−^) were added to neutralize the system.

### Molecular Dynamics Simulations

The AMBER10 program package [26] and the force filed parm03SB were chosen for simulations. The solvated systems were subjected to a thorough energy minimization and then were slowly heated form 0 up to 300 K within 600 ps with all backbone atoms constrained. Then, the non-constrained simulations were set up for 10 ns at a constant temperature of 300 K and a constant pressure of 1 atm by the Langevin piston method. The subsequent WT, N43D mutant and N43D/S138A mutant complex MD simulations were conducted as the same procedures mentioned above. The simulation time for the three complexes was prolonged to 20 ns. Altogether, five independent MD simulations were performed. During the simulations, the long-range electrostatic interactions were treated with Particle Mesh Ewald (PME) method [27], all bonds lengths involving hydrogen atoms were constrained employing the SHAKE algorithm [28], the integration time step was set to 2 fs. For three complex simulations, snapshots were extracted from the trajectory at every 1 ps from 4000 to 20000, thus yielding 8000 snapshots for each binding mode were used for the following binding energy calculation.

### Molecular Docking

Each two conformations for docking were chosen by using the agglomerative hierarchical clustering and referring to the experiment information. We aligned all receptors and ligands C34 with 6-HB of gp41, then got complex structures for docking. The conformation molecular dockings calculations were performed with the Rosettadock 2.3.0 package (versions available at http://www.rosettacommons.org). Each docking calculation generated 20000 complex structures. The final solutions were selected according to size of clusters and score. After checking the docking results, we found that all of the top 10 structures have showed the similar binding mode. The final selected complex of wild type was denoted as WT, complex of N43D mutation and C34 was denoted as N43D single mutant, while the complex of N43D mutation and C34 with S138A mutation was denoted as N43D/S138A double mutant. These three complexes were submitted to minimizations and MD simulations, the process have been addressed above in detail. After MD simulations, three complex structures were aligned with crystal structures (PDB code: 3VTP and 3VGX) [29], and calculated RMSD, the maximum RMSD between our results and crystal structures was 1.671 Å.

### MM-PBSA and MM-GBSA

Free energies of binding were estimated using the single-trajectory molecular mechanics Poisson-Boltzmann surface area (MM-PBSA) and molecular mechanics generalized Born (MM-GBSA) method in AMBER 10 *pbsa* program [30]. The binding free energies, ΔG*_b_*_(calcd)_, of a complex formed by ligand C34 and receptor were estimated as following: 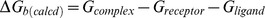


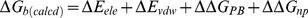
where the free energy includes electrostatic components (Δ*E_elec_*), van der Waals energy (Δ*E_vdW_*), the salvation contribution term, ΔΔ*G_solv_*, which includes two terms, the electrostatic component of the salvation free energy (ΔΔ*G_PB_*) and the nonpolar component of the salvation free energy (ΔΔ*G_np_*).

Through energy decomposition, we can analyze the contributions of key residues to the binding. Energy decomposition on per-residue is evaluated with MM-GBSA.

## Results and Discussion

### Molecular dynamics simulations

**Figure 1:**
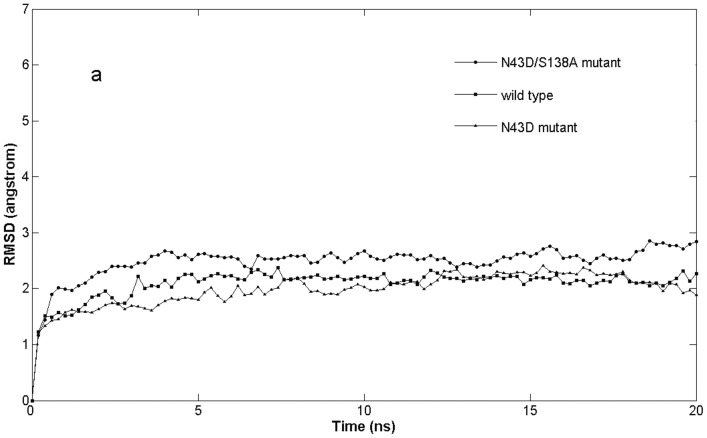
RMSD evolutions of the three models.

All fits for each system were relative to the first MD frame of the production runs and employed Cα, C, N, and O backbone atoms. The root-mean-square deviations (RMSD) of the backbone atoms with respect to the starting coordinates over the two MD simulations are plotted in Fig. 1. It can be seen clearly that during the entire period of MD the RMSD curves became flat after 5 ns, simulation runs tend to reach a plateau, indicating the complex conformations had reach equilibrium. Hence all the following analyses were performed on the last 15 ns equilibrated MD trajectories.

**Figure 2:**
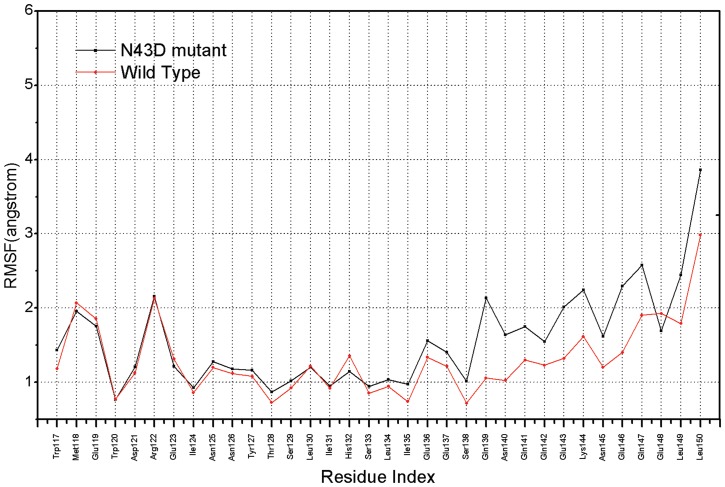
Comparisons of backbone atoms RMSF in inhibitor C34 of wild type and N43D mutant.

To track the extent of variation of individual residues of inhibitor C34 in complex, the per-residue root-mean-square fluctuations (RMSFs) of backbone atoms were computed, over the 15 ns MD simulation of inhibitor C34 in N43D mutation, we observed the 137–150 peptide of the N43D mutation type shows higher RMS fluctuations than that found in WT system. (see Fig. 2). Upon examination, we found that this is mainly owing to the charged mutation N43D, in the wild type (WT) residues in this region would interact with Asn43 of HR1 and form the Hydrogen bonds, but in the mutant type (N43D), interactions are found to be unconspicuous, the substitution of Asp for Asn breaks these interactions. The structure of these two models display different binding patterns, compare with WT system, in the mutation type, this domain of the ligand bind with receptor in a stance of torsion indicating that N43D mutation would affect the binding.

### Energentic analysis

#### Wild-type and N43D single mutant

The binding free energies were calculated by the MM/PBSA approach for snapshots collected from equilibrated trajectories of the simulation systems and are reported in Table 1. From Table 1, the contribution from electrostatic component of molecular-mechanical energy (ΔE*_ele_*) is characterized, in both two models, by relatively large positive values of ΔE*_ele_*, 169.67 kcal/mol (WT) and 459.89 kcal/mol (N43D mutant), with negative contributions to the binding free energies, ΔE*_ele_* significantly oppose complex formation. While the electrostatic solvation terms (ΔΔG*_PB_*) strongly favors the association of C34 with receptor. Although the electrostatic component of the solvation energy (ΔΔG*_PB_*) with negative values could partial compensate the severe binding energy lost of ΔE*_ele_*, overall the total electrostatic energies is negative for each model with unfavorable contribution to the final binding free energy.

**Table 1:** Binding free energies for three complexes (kcal/mol).

	Wild Type	N43D mutant	N43D/S138A mutant
*E_elec_*	169.67 (38.41)*	459.89 (34.74)*	404.88 (56.00)*
*E_vdW_*	−121.74 (6.37)*	−111.88 (7.86)*	−125.40 (7.59)*
*G_PB_*	−118.54 (36)*	−403.84 (32.65)*	−347.59 (52.79)*
*G_np_*	−14.62 (0.41)*	−14.3 (0.84)*	−15.47 (0.61)*
*G*	−85.21 (9.69)*	−70.12 (7.77)*	−83.57 (10.61)*

*standard error.

The absolute values of the van der Waals contributions (ΔE*_vdW_*) are −121.74 kcal/mol for wild type and −111.88 for N43D mutant, giving dominant contributions to the final binding free energies. The nonpolar component of solvation energy (ΔΔG*_np_*) are characterized by relatively large negative values of ΔΔG*_PB_*, −14.62 kcal/mol for wild type and −14.3 kcal/mol for N43D mutant, which have positive contributions to binding.

According to above data, we found that the contribution from each term displays a similar trend for both binding modes. The favorable formations of the complexes are mainly driven by non-polar interaction which consists of the van der Waals energy (ΔE*_vdW_*) and nonpolar contributions of solvation (ΔΔG*_np_*). And the van der Waals interactions make major contributions to binding free energy. This is in agreement with the ligand binding site of gp41 receptor which consists of a hydrophobic groove. The result is in agreement with many previous studies which have reported that hydrophobic effect is the main force for CHR and inhibitor peptide binding to gp41 [22], [31].

For N43D mutant, the contribution from ΔE*_ele_* term is negative for binding with value of 459.89 kcal/mol, which is larger than that for wild type. It should be noted that N43D introduces a negative charge on gp41 leading to repulsion between receptor and ligand. Although the polar solvation term (ΔΔG*_PB_*) contributes favorably to the binding affinity by −403.84 kcal/mol, it has partial compensate the ΔE*_ele_* term, the total electrostatic contributions is unfavorable for complex stablity. On the other hand, the dominant different between WT and N43D is ΔE*_vdW_* term.

#### N43D single mutant and N43D/S138A mutant

The calculated values of total binding free energy for formation of N43D/S138A double mutant (−83.57 kcal/mol) is more favorable than N43D single mutant (−70.12 kcal/mol). From Table 1, it comes out that the contribution from electrostatic component of molecular mechanics electrostatic energy (ΔE*_ele_*) strongly block the association of C34 with receptor, while the polar solvation term (ΔΔG*_PB_*) shows favorable contribution to binding with complex N43D/S138A. The negative value for ΔΔG*_PB_* is generally large in magnitude, which could partially compensate the energy losses of ΔE*_ele_*, so the total electrostatic energy of N43D/S138A double mutant system is similar to N43D single mutant. The absolute values of the van der Waals contributions (ΔE*_vdW_*) reach to −125.40 kcal/mol for double mutant. Similar to that observed from N43D mutant and WT system, ΔE*_vdW_* plays the dominant contribution for N43D/S138A double mutant in the binding modes between ligand and gp41. Moreover, ΔE*_vdW_* is computed to be most favorable for the N43D/S138A double mutant (−125.40 kcal/mol) compared with the systems. Overall, the energy analysis suggests that inhibitory activity of ligand C34 mimics for wild type and the C34 with S138A mutation is primarily controlled by, and best described by, van der Waals contributions (ΔE*_vdW_*), and the negative value for ΔΔG*_np_*+ ΔE*_vdW_*, which can be thought of as the intermolecular hydrophobic effect, is generally larger in magnitude than ΔΔG*_PB_*. This further suggests that hydrophobic effect dominate association.

As shown in Table 1, it comes out that the contribution of molecular mechanics electrostatic energy (ΔE*_ele_*) is quite unfavorable for both N43D single mutant and N43D/S138A double mutant with values of 459.89 kcal/mol and 404.88 kcal/mol, and the electrostatic solvation terms (ΔΔG*_PB_*), with values of −403.84 kcal/mol and −347.59 kcal/mol give compensation to the severe binding energy lost of ΔE*_ele_*. Therefore, the total electrostatic energy contribution (*PBELE = *ΔE*_elec_+*ΔΔG*_PB_*) to N43D/S138A double mutant (57.30 kcal/mol) is comparable with values of N43D single mutant (56.05 kcal/mol). Besides, the ΔΔG*_np_* term is similar between two systems. Differently, van der Waals contribution (ΔE*_vdW_*) has larger favorable contribution for N43D/S138A double mutant than single mutant. It should be noted that the main binding energy differences between N43D single mutant and N43D/S138A double mutant come from van der Waals contributions (ΔE*_vdW_*).

### Analysis of the predicted complexes

#### Wild-type and N43D mutant

**Figure 3:**
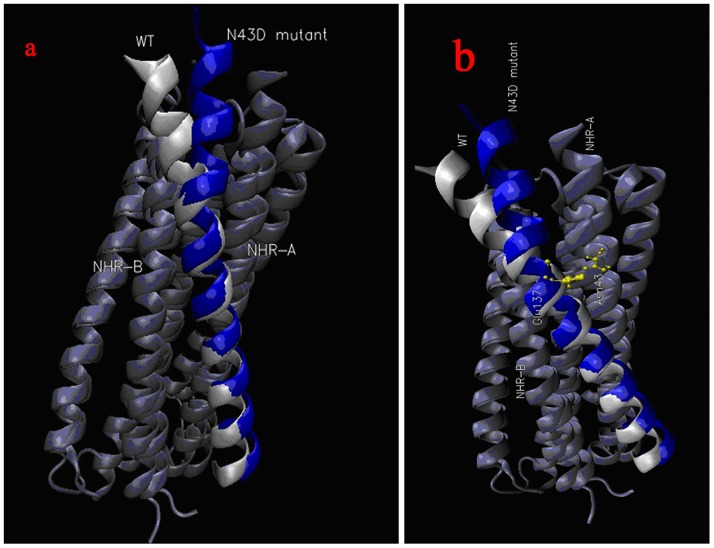
Structures of wild type (white) and N43D mutant (blue).

Structures of two models are shown in Fig. 3, wild type and N43D mutant have displayed different binding patterns. Here, for convenience, NHR chains interacting with CHR (or C34) are represented with NHR-A and NHR-B. In wild type, C34 is compressed into α-helix along the inner trimeric core of the NHR of gp41, and located at the hydrophobic groove. The binding region in N43D mutant is similar to that found in WT, while the configuration of inhibitor C34 is different, in the N43D mutant, the single mutation would cause the structure torsion of C34. We observed the binding interface of C34 in complex structures, the binding site of residues from 137 to 145 of wild type is deep inside the hydrophobic groove comparing with that in mutation type. In wild type, residues after E137 insert into the hydrophobic groove by interacting with NHR-B. However, in N43D mutant, structure torsion of inhibitor C34 would interfere with the orientation of these residues, the interaction involving these residues (residues after E137) and hydrophobic groove of NHR is changed, residues of C34 have more interactions with NHR-A.

By comparing the conformation of two systems, we believe that the change of hydrophobic interaction is resulted from the presence of structure torsion induced by electrostatic repulsion. Since the structure torsion changes the binding mode of residues 137–145 to receptor, the reduced interaction of this region of N43D mutation may interfere with the stability of binding, alterated of stability on this region mainly manifests in the higher RMS fluctuations.

#### N43D/S138A double mutant

**Figure 4:**
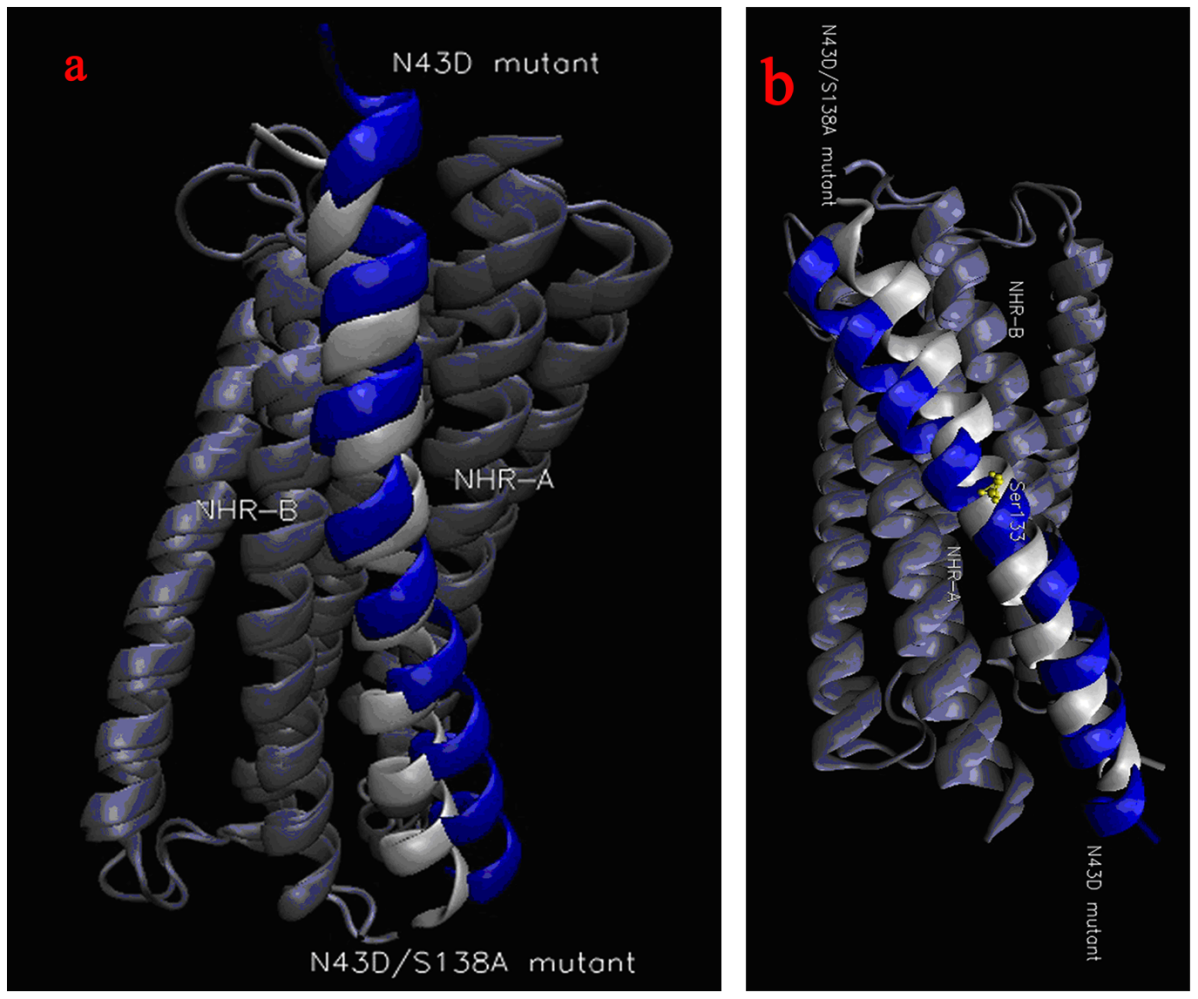
Structures of N43D mutant (blue) and N43D/S138A double mutant (white).

Analysis of double mutant crystal structures may explain the reason why double mutant is favorable for binding. Fig. 4 displayed the binding mode of mutants. When we compared the averaged structure of MD simulations, we found no evidence of a torsion emerged in double mutant. It is evident from the structure that the inhibitor C34 behaves quite differently in the N43D single mutant versus how it behaves in the resistant double mutant, and its binding mode may be more stable in the double mutant model. From the structure, in double mutant residues 117–133 of C34 has inclined to NHR-B chain of the receptor, which may make the binding more stronger. From the binding energy analysis, double mutant N43D/S138A shows more favorable binding energy than N43D single mutant, this could be due to changes as induced by the rearrangement of the binding poses of C34 during MD simulations.

### Effect of the mutation

#### N43D single mutation

**Figure 5:**
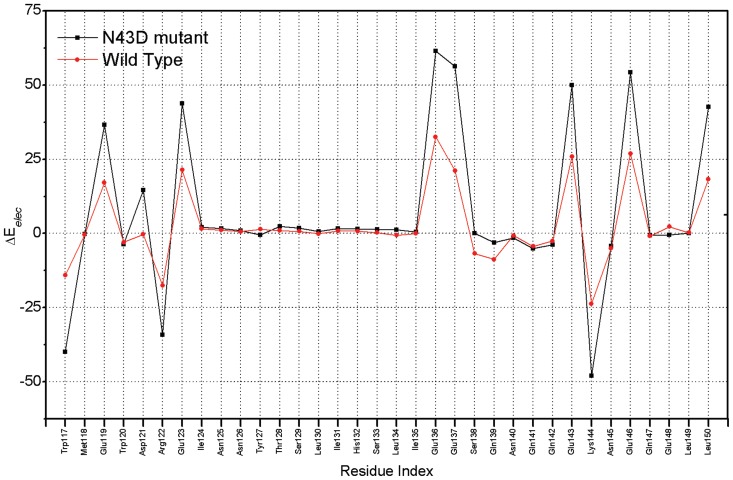
Per-residue differential in ΔE*_elec_* term for inhibitor C34 of wild type and N43D mutant (kcal/mol).

Energy decomposition enables us to observe the energetic contribution of every residue in the receptor to binding with C34. In order to identify the key residues which are important for association, the free energy decomposition based on residues was performed through MM-GBSA method. Per-residue changes in ΔE*_elec_* term were computed both in wild type and N43D mutant models. As shown in Fig. 5, residue D43 is located near all four glutamic residues of C34, thus may lead to repulsion between negatively charged residues and result in conformation changes and dramatic energies loses. Especially for residue E137, great energy losses were found from either our results (Fig. 5) or other studies [17], [32].

**Figure 6:**
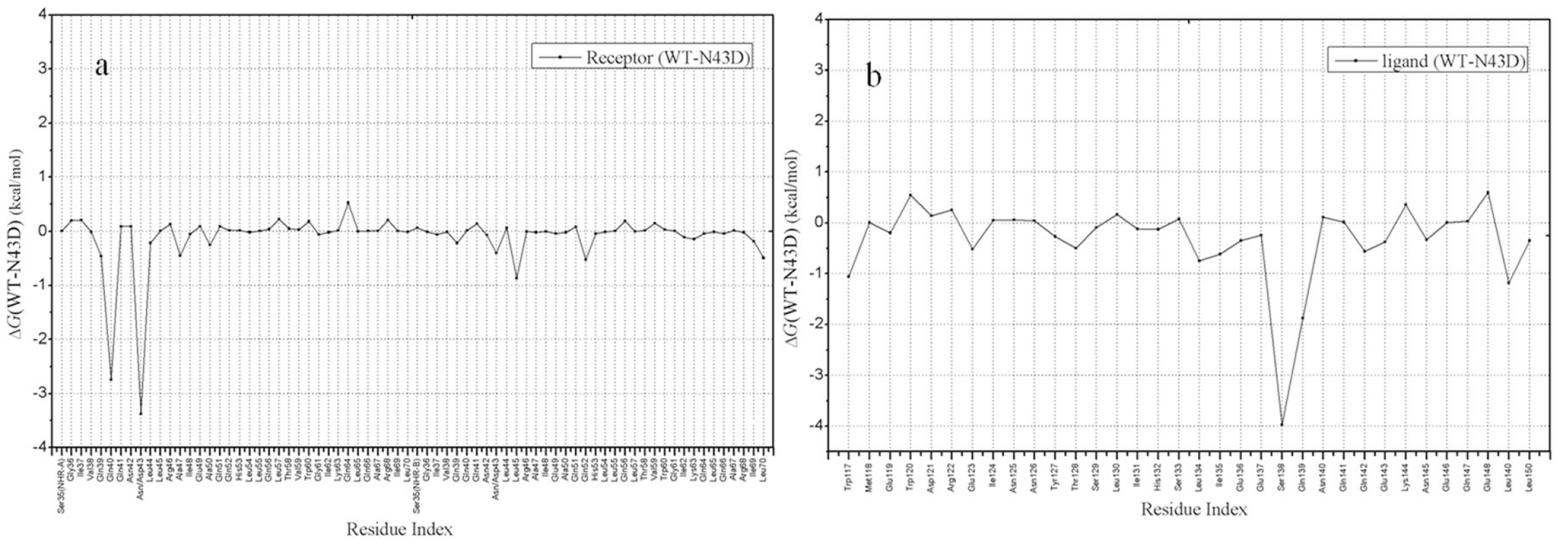
Per-residue differential in binding free energies for two system. (a) Per-residue differential (wild type minus mutant) footprints for receptor (b) Per-residue differential (wild type minus mutant) footprints for C34 (kcal/mol).

**Figure 7:**
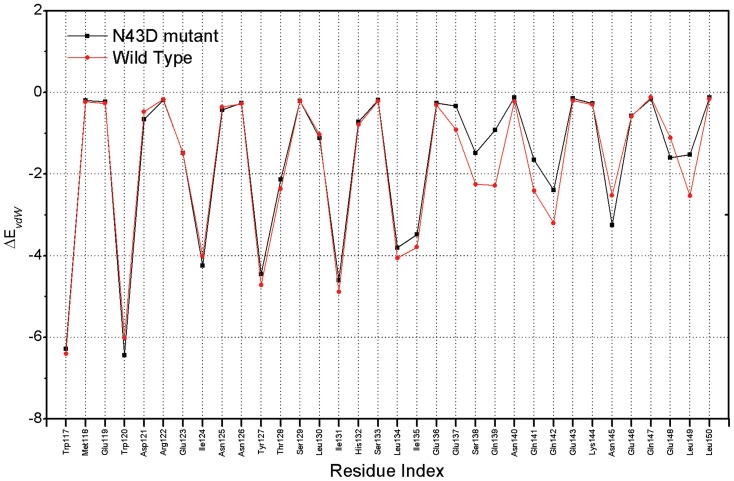
Per-residue changes of inhibitor C34 in van der Waals (kcal/mol).

Per-residue changes of binding energy in two systems are shown in Fig. 6, Per-residue differential (wild type minus mutant) footprints for receptor and ligand is shown. From Fig. 6, we can observe that in two systems (the wild type and N43D mutant), residues Q40, N/D43 and S138 have large differences for energetic contribution. As shown in Fig. 6, after the mutation N43D on NHR of gp41, Q40, N/D43 and S138 showed larger losses of energetic contribution. In fact, the energetic difference is related to the binding mode of ligand after the N43D mutation. Per-residue changes in van der Waals were computed. As shown in Fig. 7, the two curves for wild type and N43D mutant system are nearly superimoposable, except residues near the torsion. In Fig. 7, residues E137, S138, Q139, Q141 and Q142 show larger losses of van der Waals energy in N43D single mutant than wild type.

From the analysis, it could be concluded that the electrostatic repulsion is the main reason that causes the van der Waals energy losses in N43D single mutant. In fact, structure changes caused by electrostatic repulsion also have large unfavorable effects on hydrogen bonding interaction in N43D mutant.

In order to reveal differences of hydrogen bonding pattern between wild type and N43D mutant, we carried out comparative analyses of the hydrogen bonds (H-bonds) with an occupancy>30% over both complex trajectories (Table 2). In wild type, the hydrogen bond is formed between N43 and S138, E49 and Q139. Meanwhile, Q40 donates hydrogen bonds to Q142 oxygen atoms, and another hydrogen bond is formed between hydrogen atom of Q141 and the oxygen atom of Q40 in the receptor. These hydrogen bonds referred in our data are consistent with some researches, which reported that a cluster of conserved glutamine, asparagine residues (^8^QQQNN^12^) of 5-helix and (^221^QNQQ^224^) of C34 have the potential to form a hydrogen-bonded layer [22], [31], [33], [34]. But in N43D mutant, only one hydrogen bond exists, which is formed between OD2 of D43 and Q141. As previously mentioned, glutamine-rich layer has potential to form hydrogen bonds. In N43D mutant, these hydrogen bonds are collapsed, which may lead to enhanced mobility of inhibitor (C34) observed compare with wild type. Summing up the H-bonds at interface, we can see seven residues N43, S138, E49, Q139, Q40, Q141 and Q142 are involved in H-bonding in wild model, and for N43D mutation complex, only Q141 and mutation N43D are involved in hydrogen bond formation.

**Table 2:** Comparison of hydrogen bonding pairs within wild type and N43D mutant.

Wild Type		
Donor	Acceptor	occupied %
Ser138-OG-HG	Asn43-OD1	58.97
Gln139-NE2-H	Glu49-OE2	39.01
Gln141-NE2-H	Gln40-OE1	31.27
Gln40-NE2-HE	Gln142-OE1	30.68
**N43D mutant**		
Gln141-NE2-H	Asp43-OD1	41.76

From free energy analysis, it is clear that in wild type model hydrophobic interactions play crucial role in stabilizing complexes and increasing binding affinities. Moreover, the hydrogen bonding provides additional stabilizations. Combining free energy data and hydrogen bonding, the N43D mutation seems to be an unfavorable mutation to binding. The electrostatic repulsion induces large structural fluctuation (Fig. 2) thus weakens the hydrophobic interaction and effects hydrogen bonds between receptor and inhibitor C34.

#### N43D/S138A double mutation

**Figure 8:**
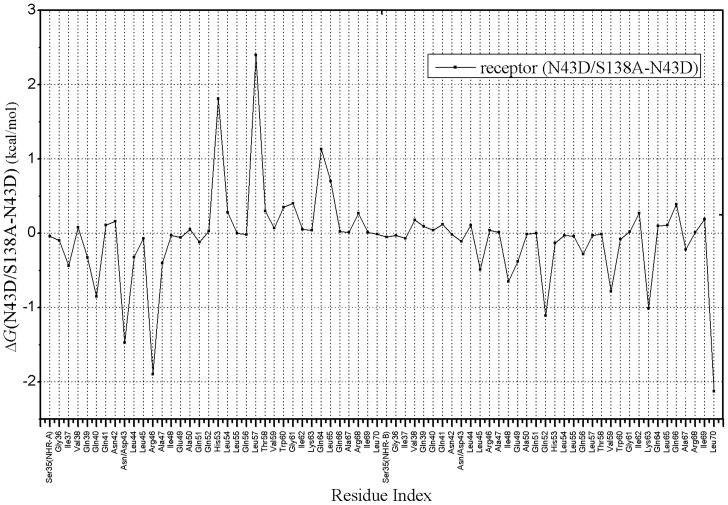
Per-residue differential (N43D/S138A double mutant minus N43D single mutant) footprints for receptor (kcal/mol).

**Figure 9:**
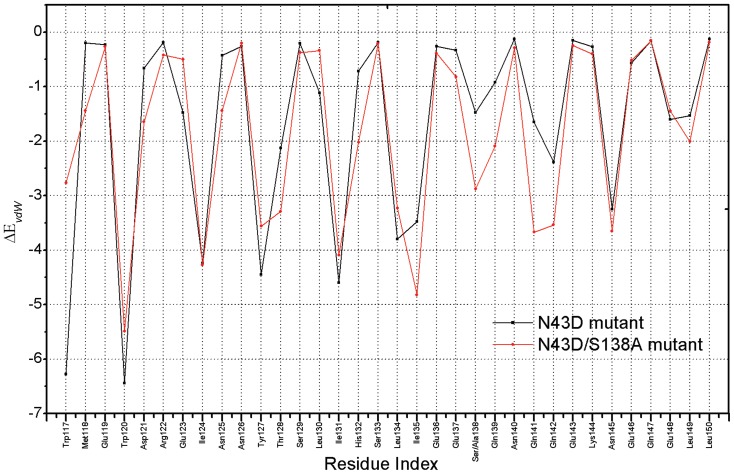
Per-residue differential in van der Waals for inhibitor C34 of N43D mutant and N43D/S138A double mutant (kcal/mol).

**Figure 10:**
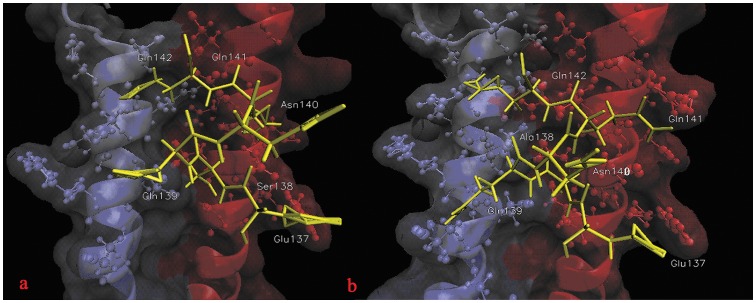
Interactions between CHR (C34) and NHR for N43D and N43D/S138A mutant. a and b show interactions between 137–145 residues of CHR (C34) and two mutants, respectively.

**Figure 11:**
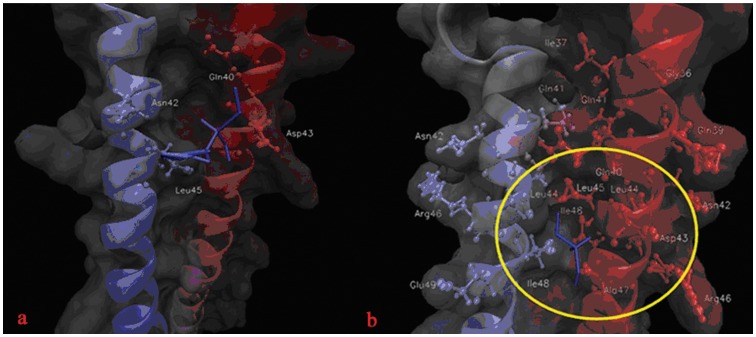
Interactions between residue S/A138 and NHR for N43D and N43D/S138A mutant. a and b represent interactions between S/A138 and NHR residues in N43D single mutant and N43D/S138A double mutant.

In order to gain extra insight into the mechanism of the fact that double mutant has greater binding energy than single mutant, and to determine hotspot regions within binding site and specific amino acids which play important roles for binding, a footprints analysis for energetic contributions is performed (Fig. 8). As displayed in Figure 4, CHR (C34) of N43D/S138A double mutant binds to NHR without structure torsion, CHR has inclined to NHR-B chain comparing with single mutant. It makes CHR of double mutant has more interactions with NHR-B, as the result, residues before S133 of CHR may make more interactions with chain NHR-B and residues after S133 may have more interactions with NHR-A (Fig. 8), with more interactions, CHR binds with receptor more stronger. Because the contribution of van der Waals interaction is the main difference between single mutant and double mutant, the footprints analysis for van der Waals interaction is explored, Fig. 9 showed the key residues in CHR (C34) which displayed great changes between two systems. It reveals that W117, W120, I124, Y127, T128, I131, L134, I135, A138, Q141 and Q142 of ligand in N43D/S138A double mutant make strong interactions with receptor. But for single mutant system, interactions for I135, A138, Q139, Q141 and Q142 are relatively weak. It had be reported that van der Waals interactions would be negligible when the distance between molecular is larger than 2.5 σ [35]. To visualize the different van der Waals contribution of these residues in two systems, the structures of inhibitor and its contact residues are determined with the default cutoff of 8.5 Å, respectively. We examined the contact residues of E137, S138A, Q139, Q141 and Q142 for two systems with cutoff of 8.5 Å. (Fig. 10a and Fig. 10b show interactions between NHR and these residues of N43D and N43D/S138A systems, respectively). Compared with single mutant, the binding mode of N43D/S138A is much favorable for binding in this region, which leads to enhanced van der Waals interactions. Fig. 10a and Fig. 10b shows that the binding interface of double mutant has induced more profit of hydrophobic interaction. It indicated that compensatory mutation S138A may enhance the binding stabilization between NHR and CHR through additional hydrophobic contacts, as many studies mentioned [36]. The crystal structure of 6HB has shown that the S138 side chain interacts with Q40 and L45 [17], [23]. We examined the structure of N43D single mutant system and found that S138 could contact with L45 and D43. Some researches suggested that A138 contacts with hydrophobic cavity formed with residues N43, L44, L45, A47 and I48 [17], in our research, the compensatory mutant A138 is optimal for binding into hydrophobic cavity formed by Q40, L44, L45, A47 and I48 (Fig. 11). Therefore, we suggest that the different behavior of C34 in two systems induce huge energetic changes, the binding mode of double mutant results in the favorable binding near the A138. This slight structure difference between single mutant and double mutant has caused these more profit of interactions at interface. By this line of reasoning, the compensatory mutation S138A (yielding N43D/S138A) induced the greater hydrophobic interactions between NHR and CHR, and may led to greater resistance to drug than the single N43D escape mutant.

The mutation S138A could explain the differential behavior of the inhibitor C34 in two systems. In single mutant, N43D that induced the electrostatic repulsion led the interaction between NHR and C34 disrupted. During the simulation, residues on C34 showed higher fluctuation. Conversely, for double mutant, C34 forms stable interactions with NHR, because of the compensatory mutation S138A, ligand C34 moves into the other chain of NHR, and occupied the hydrophobic groove with different pattern of the wild type. It is very important to note that the binding mode of N43D/S138A complex, which 1) alter the orientation of residues on the end of C34, thus allow the ligand embedded in the hydrophobic groove deeply, and stabilizes the inhibitor binding position in the complex through the increased van deer Waals interaction, 2) is involved in hydrogen bonding interactions with NHR. At the N43D single mutant complex simulation, D43 made hydrogen bonding with Q141. For the entire simulation of double mutant system, beside the interaction mentioned above, E49 make hydrogen bonding with Q139 of C34 and H53 make the H-bond with Y127.

### Hypothetical mechanism for drug resistance of gp41

As referred before, the mutation on NHR would affect the rate of membrane fusion [14], affect the infectivity of HIV-1. It has been confirmed that this mutation on NHR would delay the fusion rates in gp41 mediated cell-cell fusion [18]. Mutation on CHR, like S138A associated with the appearance of NHR N43D mutation, could restore fusion rates and show increased level resistance over than single mutant [23], [32]. S138A mutation might play a compensate role that rescue the binding between NHR and CHR [17].

All of our simulation results and observations offer us to hypothesize mechanism for drug resistance of gp41. The presence of the N43D mutation on NHR could affect the binding between inhibitor C34 and NHR. The mutation N43D affects the conformation of the residues from 137 to 145 on inhibitor C34 far away from the hydrophobic grooves, and the electrostatic repulsion would affect the binding stability of inhibitor, thus might reduce the efficiency of inhibitor. However, the conformation change of gp41 has been carried out necessary for membrane fusion. When mutation N43D prevents the binding of inhibitor, it would have impact on the binding of the CHR domain as well, and lead to dramatic changes in the NHR-CHR interface. This defect observed in N43D single mutation may obstruct the stability of 6-HB conformation, thereby may disturb the membrane fusion and the fusion rates.

When the N43 and S138 residues are mutated to aspartic and alanine, the S138A mutation might affect the conformation of CHR near the hydrophobic grooves that adopts a more constrained conformation with the residues before S133 pointing in the opposite direction of the NHR. Because of CHR move into the hydrophobic groove region deeply, CHR domain would make more hydrophobic interaction and hydrogen bonds with NHR. In our simulation results, N43D/S138A double mutant displayed stronger binding energy than N43D, S138A mutation might compensate for the binding between NHR and CHR.

At this point we hypothesize that the compensatory mutation S138A (yielding N43D/S138A) would induce the greater hydrophobic interactions between NHR and CHR, enhance the binding of CHR and NHR, and in contrast to this, the binding affinity between the inhibitor and gp41 is reduced. This binding mode of double mutant seems to be an acceptable model that eliminates the fitness defect of HIV-1 that caused by N43D mutation on NHR.

## Conclusions

Many researches and intensive studies have recently offered facilities for the HIV drug design and mechanism of drug resistance [37], [38]. In this work, we focused on the two mutants of gp41, N43D single mutation and compensatory mutation S138A (yielding N43D/S138A), and delineated detailed characteristics of the interactions and binding model on two mutants. Detailed analyses of structural alteration in complexes indicate that the inhibitor C34 displays different binding modes in wild type and two mutants.

It is found that the N43D mutation introduced a negative charge on receptor, thus led to repulsion between receptor and ligand, and induced the conformation changes of complex, and led to greater losses in hydrophobic interactions and hydrogen bond interaction. Results showed that the N43D mutation is detrimental for inhibitor C34 binding, as many studies mentioned that fusion inhibitors are susceptible to resistance mutations [13], [24], [39]–[41]. Interestingly, as mentioned above, N43D mutation on NHR affects the membrane fusion mediated by gp41. In our study, binding affinity of CHR (C34) to NHR with N43D mutation is diminished. These results may demonstrate once again that neither inhibitor nor HIV-1 itself would escape the impact of N43D single mutation.

Conversely, the MD simulation of N43D/S138A double mutant displays a different binding mode between CHR (C34 with S138A mutation) and NHR (with N43D mutation). This mode let CHR have more contact with hydrophobic residues of receptor, induced more van der Waals contributions for binding. This great hydrophobic interaction of double mutant could partially offset the energy loss arising from N43D. The change of binding mode on N43D/S138A identified here could help to explain why compensatory mutation S138A could partially restores infectivity, lead to increased infectivity and greater resistance to inhibitors than mutation N43D.

To understand the drug resistance of HIV-1 fusion inhibitors is important for novel medicine design. The reasonable hypothetical mechanism of this work has provided insights into the detailed mechanism of drug resistance, especially the double mutation of gp41.
